# Assessing unmet needs in advanced cancer patients: a systematic review of the development, content, and quality of available instruments

**DOI:** 10.1007/s11764-021-01088-6

**Published:** 2021-08-06

**Authors:** Ben Rimmer, Lisa Crowe, Adam Todd, Linda Sharp

**Affiliations:** 1 Population Health Sciences Institute, Newcastle University, Newcastle University Centre for Cancer, Newcastle, England; 2 School of Pharmacy, Newcastle University, Newcastle, England

**Keywords:** Cancer, Advanced disease, Unmet needs, Instrument

## Abstract

**Purpose:**

Advances in treatment, including biological and precision therapies, mean that more people are living with advanced cancer. Supportive care needs likely change across the cancer journey. We systematically identified instruments available to assess unmet needs of advanced cancer patients and evaluated their development, content, and quality.

**Methods:**

Systematic searches of MEDLINE, CINAHL, Embase, PubMed, and PsycINFO were performed from inception to 11 January 2021. Independent reviewers screened for eligibility. Data was abstracted on instrument characteristics, development, and content. Quality appraisal included methodological and quality assessment, GRADE, feasibility, and interpretability, following consensus-based standards for the selection of health measurement instruments (COSMIN) guidelines.

**Results:**

Thirty studies reporting 24 instruments were identified. These were developed for general palliative patients (n = 2 instruments), advanced cancer (n = 8), and cancer irrespective of stage (n = 14). None focused on patients using biological or precision therapies. The most common item generation and reduction techniques were amending an existing instrument (n = 11 instruments) and factor analysis (n = 8), respectively. All instruments mapped to ≥ 5 of 11 unmet need dimensions, with Problems and Needs in Palliative Care (PNPC) and Psychosocial Needs Inventory (PNI) covering all 11. No instrument reported all of the COSMIN measurement properties, and methodological quality was variable.

**Conclusions:**

Many instruments are available to assess unmet needs in advanced cancer. There is extensive heterogeneity in their development, content, and quality.

**Implications for Cancer Survivors:**

Given the growth of precision and biological therapies, research needs to explore how these instruments perform in capturing the needs of people using such therapies.

**Supplementary Information:**

The online version contains supplementary material available at 10.1007/s11764-021-01088-6.

## Introduction

For many cancers, survival continues to improve [1, 2]. This progress is, in part, due to medical advancements, such as improved diagnostic techniques and more effective treatment strategies – examples of which include precision and biological therapies [3, 4]. These agents are primarily – although not exclusively – used for patients with advanced disease. While they may improve survival, they are associated with adverse effects (e.g. cardiac dysfunction, hypertension, and skin rashes [5, 6]), which are different to those associated with traditional chemotherapeutic treatments. Patients using these new therapies may therefore experience a different symptom burden. Changes in advanced cancer prognosis, largely due to the growth in these therapies, mean that the cancer experience, once typified by rapid progression, may now be a prolonged and uncertain illness trajectory [7].

Previous work has identified a comprehensive range of unmet supportive care needs among advanced cancer patients [8, 9]. The most common dimensions of unmet need are physical, psychological, informational, and functional, with others including social, activities of daily living, healthcare, spiritual, sexual, and economic also described. People with early or advanced cancer may experience similar areas of unmet need. However, the prevalence, severity, and relative distribution of needs may vary between the two patient groups. This could result from location of the cancer (e.g. metastatic disease may result in more pain and greater physical unmet needs), different treatment (e.g. the differing adverse effect profiles of biological and precision therapies, compared to traditional chemotherapy), and prognosis (e.g. psychological unmet needs among patients with advanced disease may be dominated by emotions and worries about coming to the end of life). In terms of consequences, unmet needs in people with advanced cancer have been associated with more symptom distress, greater anxiety, and reduced quality of life [9]. Supportive care that is not consistent with patient needs could be detrimental to the patient, their caregiver, and even healthcare expenditure [10, 11].

Despite many examples of needs assessment tools and instruments available for use in people with cancer [12], it is not known which, if any, specifically capture the needs of people with advanced cancer. As supportive care needs change across the cancer journey [13], establishing what instruments are available to assess the needs of people with advanced cancer must be understood. This is particularly important when you consider the unique challenges presented by the new biological and precision therapies in this context. Our systematic review, therefore, aimed to address this question and (1) examine what instruments are available to measure unmet needs in people with advanced cancer and (2) assess instrument development, content, and quality, in terms of clinimetric properties.

## Methods

This systematic review was registered with the Prospective Register for Systematic Reviews (PROSPERO) (CRD42020169278) and conducted and reported in accordance with the Preferred Reporting Items for Systematic Review and Meta-Analysis (PRISMA) guidelines [14].

### Definitions

There are multiple ways in which ‘advanced cancer’ can be defined. For the purposes of this systematic review, advanced disease was considered to be patients with stage IV, metastatic or incurable disease, or those undergoing palliative care.

An ‘unmet need’ was defined as something that a patient experiences as a problem *and* which they would like help or support with.

### Eligibility criteria

A study was eligible if: (1) it reported on the development and/or validation of an instrument to measure unmet needs; (2) it included or signposted to the instrument items; (3) advanced cancer patients were included in the development or validation of the instrument; and (4) it was an original article, available in English. The instrument that a study pertains to was eligible if: (1) it was developed for cancer or palliative patients; (2) it measured more than one dimension of unmet need; and (3) it was available in English.

A study was excluded if: (1) the instrument was targeted at childhood/adolescent cancer patients, or survivors of cancer diagnosed in childhood/adolescence; (2) the authors did not report any validation for the instrument; (3) the patient was not the respondent; and (4) ≥ 50% of the instrument items and response options did not either allow patients to indicate a desire for help or support or use terminology that could infer a need/desire for help or support.

### Search strategy

Five electronic databases were searched from inception: MEDLINE, Embase, PsycINFO, CINAHL, and PubMed. The search strategy concerned four key concepts (cancer, advanced disease, needs, and instrument), and was undertaken in March 2020. A combination of medical subject headings and keywords was formulated, with assistance from a senior library assistant (*Online Resource* *1*), and informed by previously published search strategies [8]. Searches were tailored in accordance with the specific subject headings within each database (*Online Resource* *2*).

The reference lists and forward citations of eligible studies and relevant systematic reviews were handsearched to identify additional studies. The search was updated on 11 January 2021, with no new studies identified.

### Study selection

After duplicate studies were removed, titles and abstracts and then full-texts of potentially eligible studies were independently screened by two researchers (BR and LC). Disagreements were resolved through discussion and consensus with other authors, if required. Where cancer stage of study participants was not reported, authors were contacted to confirm inclusion of advanced cancer patients in the instrument’s development or validation. If eligibility was not confirmed, the instrument was excluded. If an existing instrument was adapted or shortened, with separate validation, this was included as a separate instrument. If an existing instrument was refined (e.g. item wording was modified), only the refined version was included.

### Data extraction

#### General characteristics of included instruments

Data extraction was undertaken by BR, following a structured data extraction form, and checked by LS and AT. Extracted data included instrument name; purpose; target population; validations in languages other than English; study setting; study population; number of questions; mode of administration; recall period; time to complete; scoring; response options; item generation (e.g. patient interviews) and reduction (e.g. item response frequencies); and unmet need dimensions measured. Disagreements were resolved through discussion and consensus.

Published papers reporting additional development and/or validation studies relating to the included instruments were identified and used in data extraction where relevant. Where multiple papers were available for an instrument, characteristics of the study population were extracted from the initial (first published) validation study; all relevant papers were used in the final instrument development and content, and findings were pooled across papers for assessment of clinimetric properties.

#### Content analysis

Instrument content was mapped against nine previously identified dimensions of unmet need [8, 9]. Two additional dimensions, autonomy and role, were added based on the content of the eligible instruments (*Online Resource* *3*). Any content that did not map onto these dimensions was reported as ‘other’.

Instrument items were included in this mapping if the items and/or response options allowed respondents to indicate a desire for help or difficulty with the item, thus signifying or inferring an unmet need. Dimension development was categorised as statistical (e.g. factor analysis), literature, conceptual, or other.

#### Clinimetric properties

The COSMIN checklist [15, 16] (*Online Resource* *4*) was used to assess the methodological quality of the included instruments. COSMIN evaluates the development, validity, reliability, and responsiveness of instruments. The checklist is divided into ten measurement properties: development, content validity, structural validity, hypotheses testing, internal consistency, reliability, measurement error, cross-cultural validity, criterion validity, and responsiveness. Cross-cultural validity was not measured in this review because clinimetrics were only assessed in the original English language versions of the instruments. Criterion validity was omitted because there is no gold standard for needs assessments, due to the subjective nature of perceived needs [17].

#### Methodological quality assessment

The evaluation of each clinimetric property comprised 3–35 items. Each item is rated on a four-point scale: *very good*, *adequate*, *doubtful*, or *inadequate*. In accordance with ‘the worst score counts’ principle of COSMIN [15], the lowest score within a measurement property determined the methodological quality rating given to the instrument for that property.

GRADE was used to summarise the quality of available evidence. This concerned risk of bias, inconsistency, imprecision, and indirectness. Evidence was downgraded appropriately (*Online Resource* *5*) and could be *high* (†††), *moderate* (††), *low* (†), or *very low* (-) in quality.

#### Quality criteria for the measurement properties

The quality of six measurement properties was assessed using a three-point scale: *sufficient* (+), *insufficient* (-), and *indeterminate* (?). This applied to structural validity, internal consistency, reliability, measurement error, hypotheses testing for construct validity, and responsiveness.

#### Feasibility and interpretability

To inform usability, instrument feasibility and interpretability were extracted. Feasibility within COSMIN concerns the ease of applying the instrument in its intended context of use, so aspects including instrument length, completion time, and type and ease of administration were extracted. Interpretability concerns the extent to which meaning can be assigned to quantitative scores, so aspects including distribution of scores and percentage of missing items and missing total scores were extracted.

## Results

### Search results

**Fig. 1:**
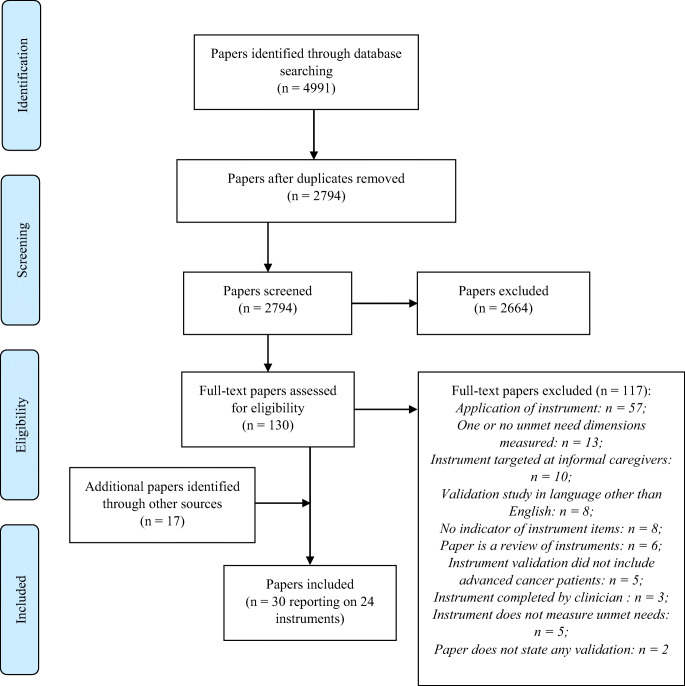
PRISMA flow diagram of study selection

The database searches identified 4991 hits, with 2794 remaining after deduplication. After title and abstract screening, 130 full-text articles were assessed for eligibility, and of these, 13 studies were eligible. Following hand searching, an additional 17 studies were identified and deemed eligible. Overall, 30 papers reporting on 24 unique instruments were included in the review (*Fig.* *1*).

### Study population for validation

Of the 24 instruments, two were ‘general palliative’ instruments (Patient Needs Assessment in Palliative care (PNAP) [18]; Sheffield Profile for Assessment and Referral for Care (SPARC) [19]) developed in mixed samples of people with advanced illnesses (including cancer). Eight were ‘advanced cancer’ instruments (Needs Assessment for Advanced Cancer Patients (NA-ACP) [17]; Needs Assessment for Advanced Lung Cancer Patients (NA-ALCP) [20]; Problems and Needs in Palliative Care Questionnaire (PNPC) [21]; Problems and Needs in Palliative Care Questionnaire–short version (PNPC-sv) [22]; Screen for Palliative and End-of-Life Care Needs in the Emergency Department (SPEED) [23]; Three Levels of Needs Questionnaire (3LNQ) [24]; name not given (Ndiok) [25]; Palliative Care Needs Assessment–English/Arabic Versions (PCNA-EAV) [26]). Fourteen were ‘all stage cancer’ instruments validated in a sample that included people with advanced cancer (Cancer Needs Distress Inventory (CaNDI) [27]; Comprehensive Needs Assessment Tool in Cancer (CNAT) [28, 29]; Psychosocial Needs Inventory (PNI) [30]; Electronic Holistic Needs Assessment (eHNA) [31]; 34-item Supportive Care Needs Survey (SCNS-SF34) [32]; Supportive Care Needs Assessment Tool for Indigenous People (SCNAT-IP) [33, 34]; Supportive Care Needs Survey–9-item Screening Tool (SCNS-ST9) [35]; 59-item Supportive Care Needs Survey (SCNS-LF59) [36]; Needs Evaluation Questionnaire (NEQ) [37–39]; Cancer Needs Questionnaire-Short Form (CNQ-sf )[40]; CancerSupportSource (CSS-25) [41, 42]; Bladder Cancer Needs Assessment Survey (BCNAS-32) [43]; Prostate Cancer Needs Questionnaire Version 2 (PCNQ V2) [44, 45]; You, Your family, and City of Hope are a team (YYFcore03) [46]). From these 14, 12 instruments were developed in a mixed sample of cancers, while the BCNAS-32 [43] and PCNQ V2 [45] were specifically developed for bladder and prostate cancer, respectively (*Online Resource* *6*).

Eight instruments were developed in Australia [17, 20, 32, 33, 35, 36, 40, 44], five in the USA [23, 27, 41, 43, 46], three in the UK [19, 30, 31], two in Netherlands [21, 22], and one in each in Czech Republic [18], Denmark [24], Nigeria [25], Saudi Arabia [26], Republic of Korea [28], and Italy [37].

### *Instrument characteristics*

Instrument length ranged from 9 to 138 items (*Table* *1*). Seventeen were self-administered [17, 20–22, 24, 27, 30–32, 35–37, 40, 41, 43, 44, 46], and seven were interviewer-administered (e.g. by a clinician) [18, 19, 23, 25, 26, 28, 33]. For the 13 instruments that reported completion time, it ranged from 5 to 76 min [17–19, 22, 26, 27, 31–33, 36, 37, 41, 44]. Patients were asked to recall their needs during the following time periods: past 4 months (NA-ACP) [17], 1 month (n = 7 instruments) [19, 26, 28, 32, 33, 35, 36], few weeks (PNI) [30], 2 weeks (CaNDI) [27], 1 week (n = 2) [18, 24], and the present day (n = 2) [41, 44]. Ten instruments did not specify a recall period [20–23, 25, 31, 37, 40, 43, 46].

**Table 1:** Instrument characteristics

**Instrument**	**Target population**	**Number of items**	**Mode of administration**	**Recall time**	**Time to complete (minutes)**	**Scoring**	**Response options**
Palliative Needs Assessment in Palliative care (PNAP) [18]	Palliative - general	40	Not reported	Past week	45	Total and Individual items - Unmet need is important (importance: 4-5) and unmet (satisfaction: 1-2)	Importance of item: 5 point scale, 1 (not at all) to 5 (very much); Whether item has been met: 5 point scale, 1 (not at all) to 5 (yes, very much)
Sheffield Profile for Assessment and Referral for Care (SPARC) [19]	Palliative - general	46	Interviewer-administered	Past month	15 to 20	Subscales and individual items	4 point scale: not at all (= 0), a little bit (= 1), quite a bit (= 2), very much (= 3);Talk to/information about the following? Yes/No
Needs Assessment for Advanced Cancer Patients (NA-ACP) [17]	Cancer - advanced	132	Self-administered	Past four months	76	Subscales	5 point scale: no need, not applicable (= 0), no need, satisfied (= 0), low need (= 1), moderate need (= 2), high need (= 3)
Needs Assessment for Advanced Lung Cancer Patients (NA-ALCP) [20]	Cancer - advanced (lung)	38	Self-administered	Not reported	Not reported	Subscales	4 point scale: no need, low need, moderate need, and high need
Problems and Needs in Palliative Care questionnaire (PNPC) [21]	Cancer - advanced	138	Self-administered	Not reported	Not reported	Subscales or individual items	Is this a problem?' 3 point scale: 'Yes', 'Somewhat', 'No'; 'Do you want professional attention for this?' 3 point scale: 'Yes, more', 'As much as now', 'No'
Problems and Needs in Palliative Care questionnaire – short version (PNPC-sv) [22]	Cancer - advanced	33	Self-administered	Not reported	10	Individual items	Is this a problem?' 3 point scale: 'Yes', 'Somewhat', 'No'; 'Do you want professional attention for this?' 3 point scale: 'Yes, more', 'As much as now', 'No'
Screen for Palliative and End-of-life care needs in the Emergency Department (SPEED) [23]	Cancer - advanced	13	Interviewer-administered	Not reported	Not reported	Individual items	11 point scale from 0 (not at all) to 10 (a great deal)
Three Levels of Needs Questionnaire (3LNQ) [24]	Cancer - advanced	35	Self-administered	Past week	Not reported	Subscales	Problem intensity & burden: 4 point scale: not at all (= 1), a little (= 2), quite a bit (= 3), very much (= 4); Felt need: have you received help (Yes/No); How adequate has this help been (inadequate/ partly inadequate/ adequate); Would you be interested in help (Yes/No)
Name not given (Ndiok) [25]	Cancer - advanced	104	Interviewer-administered	Not reported	Not reported	Individual items	Yes/No
Palliative Care Needs Assessment – English/ Arabic Versions (PCNA-EAV) [26]	Cancer - advanced	116	Interviewer-administered	Past 4 weeks	40	Subscales and individual items	Three 5 point scales, from: 1 (strongly disagree) to 5 (strongly agree), 1 (none of the time) to 5 (all of the time), 1 (extremely important) to 5 (not at all important)
Cancer Needs Distress Inventory (CaNDI) [27]	Cancer - all stages	39	Self-administered	Past 2 weeks	8	Total and subscales	5 point scale: not a problem (= 1), mild problem (= 2), moderate problem (= 3), severe problem (= 4), very severe problem (= 5);Follow-up question on whether would like to discuss concern with health professional - Yes/Prefer not to.
Comprehensive Needs Assessment Tool in cancer (CNAT) [28, 29]	Cancer - all stages	59	Interviewer-administered	Past month	Not reported	Total	4 point scale: no need (= 0), low need (= 1), moderate need (= 2), high need (= 3)
Psychosocial Needs Inventory (PNI) [30]	Cancer - all stages	48	Self-administered	Past few weeks	Not reported	Subscales	How important' 5 point scale from 1 (not important) to 5 (very important);'How satisfied' 5 point scale from 1 (not satisfied) to 5 (very satisfied)
electronic Health Needs Assessment (eHNA) [31]	Cancer - all stages	48	Self-administered (touchpad)	Not reported	7	Total and individual items	11 point scale from 0 (no problem) to 10 (maximum concern)
34-item Supportive Care Needs Survey (SCNS-SF34) [32]	Cancer - all stages	34	Self-administered	Past month	10	Subscales	5 point scale: no need, not applicable (= 1), no need, satisfied (= 2), low need (= 3), moderate need (=4), high need (= 5)
Supportive Care Needs Assessment Tool for Indigenous People (SCNAT-IP) [33, 34]	Cancer - all stages	27 (26 + 1 open-ended)	Interviewer-administered	Past month	15	Total and subscales	Yes or no, then if answered yes, 4 point scale: satisfied with help received (=1); needed a little more help (=2); needed some more help (=3); needed a lot more help (=4)
Supportive Care Needs Survey – 9-item Screening Tool (SCNS-ST9) [35]	Cancer - all stages	9	Self-administered	Past month	Not reported	Individual items	5 point scale: no need, not applicable (= 1), no need, satisfied (= 2), low need (= 3), moderate need (=4), high need (= 5)
59-item Supportive Care Needs Survey (SCNS-LF59) [36]	Cancer - all stages	59	Self-administered	Past month	20	Subscales	5 point scale: no need, not applicable (= 1), no need, satisfied (= 2), low need (= 3), moderate need (=4), high need (= 5)
Needs Evaluation Questionnaire (NEQ) [37–39]	Cancer - all stages	23	Self-administered	Not reported	5	Subscales and individual items	Yes or No
Cancer Needs Questionnaire – short form (CNQ-sf) [40]	Cancer - all stages	32	Self-administered	Not reported	Not reported	Subscales	5 point scale: no need, not applicable (= 1), no need, satisfied (= 2), low need for help (= 3), moderate need for help (=4), high need for help (= 5)
CancerSupportSource (CSS-25) [41, 42]	Cancer - all stages	25	Self-administered	Today	5 to 10	Total and depression and anxiety scores	5 point scale rating level of concern: not at all (=0), slightly (=1), moderately (=2), seriously (=3), very seriously (=4);Prompted with each item, "Please let us know how we can help you…" - select all that apply: Have a staff person talk with you; Connect you with online resources; Give you written information; or No action needed
Bladder Cancer Needs Assessment Survey (BCNAS-32) [43]	Cancer - all stages (bladder)	32	Self-administered	Not reported	Not reported	Subscales	5 point scale from 1 (no need, not applicable) to 5 (high need).
Prostate Cancer Needs Questionnaire Version 2 (PCNQ V2) [44, 45]	Cancer - all stages (prostate)	69 (pt.1 39, pt.2 30)	Self-administered	Part 1: at time of diagnosis/ treatment decision; Part 2: Now	30	Individual items	5 point scale from 1 (Strongly disagree) to 5 (Strongly agree) for extent of problem, and 5 point scale for level of help that would be/have been desirable: None at all, A little, Some, Quite a bit, A lot
You, Your family, and City of Hope are a team (YYFcore03) [46]	Cancer - all stages	31	Self-administered (touchpad)	Not reported	Not reported	Individual items	7 point scale for extent of problem from 1 (Not a problem) to 5 (Very severe problem), Prefer not to answer and Do not know; If moderate, severe, or very severe problem, an in person follow up will occur. If any other response: "How can we best work with you on this problem?": Nothing needed at this time, Talk with a member of the team, Provide written information, Written information and talk with team member.

Fourteen instruments calculated subscale scores [17, 19–21, 24, 26, 27, 30, 32, 33, 36, 37, 40, 43], 12 scored each individual item [18, 19, 21–23, 25, 26, 31, 35, 37, 44, 46], and six calculated total scores [18, 27, 28, 31, 33, 41]. Eight instruments calculated more than one score type [18, 19, 21, 26, 27, 31, 33, 37]. Fourteen instruments asked patients to indicate – often through 4- or 5-point scales – the extent to which an item is a problem and/or the level of help needed [17, 18, 20, 23, 26, 28, 30–32, 35, 36, 40, 43, 44]. Four instruments used a dichotomous format to indicate (1) whether there is a problem and (2) whether they want help [21, 22, 25, 37]. Six instruments used a combination of these approaches, to indicate whether there is a problem, then how much help is needed, or vice versa [19, 24, 27, 33, 41, 46].

Eight instruments have been validated in languages other than English (*Online Resource* *7*) [19, 22, 27, 28, 32, 35, 37, 40].

### Instrument development

*Table* *2* details the item generation and reduction techniques used to develop each instrument. *Online Resource* *8* specifies the ‘other’ techniques. *Online Resource* *9* signposts to qualitative papers that informed an instrument’s development.

**Table 2:** Item generation and reduction techniques used in instrument development

Instrument	Item generation								Item reduction					
	*Literature*	*Review of content of existing instruments*	*Patient discussions*^*a*^	*Expert panel*	*Conceptual model*	*Previous instrument*	*Health professional discussions*^*a*^	*Other*	*Process of item reduction reported?*	*Factor analysis*^*b*^	*Item-response frequencies*	*Patient review*	*Expert review*	*Other*
*PNAP*	+	+					+		Yes		+		+	
*SPARC*	+		+	+			+		Yes					+
*NA-ACP*	+		+				+		Yes	+				
*NA-ALCP*				+		+			Yes	+	+			+
*PNPC*		+	+	+			+	+	Yes		+			
*PNPC-sv*						+			Yes		+			
*SPEED*		+		+		+			No					
*3LNQ*	+	+				+			No					
*Ndiok*	+				+				No					
*PCNA-EAV*	+	+		+				+	No					
*CaNDI*	+		+		+		+		Yes			+		+
*CNAT*		+	+						Yes			+	+	
*PNI*	+		+						No					
*eHNA*									No					
*SCNS-SF34*						+			Yes	+				+
*SCNAT-IP*			+			+		+	Yes	+		+		+
*SCNS-ST9*						+			Yes	+	+			+
*SCNS-LF59*						+			Yes		+		+	
*NEQ*			+						Yes					+
*CNQ-sf*						+			Yes	+				
*CSS-25*			+						Yes	+	+			+
*BCNAS-32*	+		+	+					Yes		+			
*PCNQ V2*						+			Yes		+			
*YYFcore03*	+					+		+	No					

^a^Discussions refers to interviews and focus groups

^b^Factor analysis pertains to exploratory and confirmatory factor analysis, principal component analysis, and item factor loadings.

### Item generation

Fifteen instruments used more than one item generation technique [17–21, 23–28, 30, 33, 43, 46] – the most common of which were amending items from an existing instrument (n = 11 instruments) [20, 22–24, 32, 33, 35, 36, 40, 44, 46]; using the literature (n = 10) [17–19, 24–27, 30, 43, 46]; and patient interviews and focus groups (n = 10) [17, 19, 21, 27, 28, 30, 33, 37, 41, 43]. Less common techniques included review of content of existing instruments (n=6) [18, 21, 23, 24, 26, 28]; consulting with an expert panel (n = 6) [19–21, 23, 26, 43]; health professional interviews and focus groups (n = 5) [17–19, 21, 27]; and informed by a conceptual model (n = 2) [25, 27].

### Item reduction

Seventeen instruments reported an item reduction process [17–22, 27, 28, 32, 33, 35–37, 40, 41, 43, 44]. Eleven instruments reported more than one item reduction technique [18–20, 27, 28, 32, 33, 35–37, 41]. The most common techniques were factor analysis (n = 8 instruments) [17, 20, 32, 33, 35, 41, 43, 44]; item response frequencies (n = 6) [18, 20–22, 35, 40]; patient review (n = 5) [27, 28, 33, 36, 41]; expert review (n = 3) [18, 28, 36]; and test-retest reliability (n = 2) [20, 37].

*Instrument content*

The most frequently measured unmet need dimensions were psychological (n = 24 instruments) [17–28, 30–33, 35–37, 40, 41, 43, 44, 46]; healthcare (n = 22) [17–21, 23–25, 27, 28, 30–33, 35–37, 40, 41, 43, 44, 46]; activities of daily living (n = 21) [17–28, 30–33, 36, 37, 40, 41, 46]; and physical (n = 21) [17–24, 26–28, 30–33, 35–37, 40, 41, 46]. Further frequently measured dimensions comprised information (n = 19) [17–22, 25, 26, 28, 30, 32, 33, 35–37, 40, 43, 44, 46]; social (n = 18) [17–25, 27, 28, 30, 31, 37, 40, 41, 43, 44]; and sexual (n = 17) [19, 21, 22, 24–27, 30–33, 35, 36, 41, 43, 44, 46].

Of the included instruments, the PNPC [21] and PNI [30] were the most comprehensive, measuring all 11 unmet need dimensions, while CaNDI [27] measured 10. According to our criteria, SPEED [23] was the least comprehensive, measuring only five of the dimensions (*Table* *3**, Online Resource* *10*).

**Table 3:** Unmet need dimensions measured by each instrument^a,b^

**Instrument**	*Physical*	*Psychological*	*Information*	*Social*	*Activities of daily living*	*Health care*	*Spiritual*	*Sexual*	*Economic*	*Autonomy*	*Role*	*Other*
*PNAP*	+	+	*	+	*	+	+		*	+		
*SPARC*	+	+	+	+	+	+	+	*		+		
*NA-ACP*	+	+	+	+	+	*	+		+			
*NA-ALCP*	+	+	+	+	+	*	+		+			
*PNPC*	+	+	+	+	+	+	+	*	+	+	+	
*PNPC-sv*	+	+	+	+	+		+	*	+	+		
*SPEED*	+	+		+	*	+						
*3LNQ*	+	+		+	+	+		+			+	
*Ndiok*		+	+	+	+	+	+	*	+	+		
*PCNA-EAV*	+	+	+		+		+	*		*	*	
*CaNDI*	+	+		+	+	+	*	*	*	*	*	
*CNAT*	+	+	+	+	*	+	+		*		*	
*PNI*	*	+	+	+	+	+	+	*	*	*	*	Identity
*eHNA*	+	+		+	+	+	+	+	*		+	
*SCNS-SF34*	+	+	+		+	+		+		*		
*SCNAT-IP*	+	+	+		+	+		+	*	*		
*SCNS-ST9*	+	+	+			+		+		*		
*SCNS-LF59*	+	+	+		+	+		+	*	*		
*NEQ*	*	+	+	+	+	+	+		+			
*CNQ-sf*	+	+	+	+	+	*				*		
*CSS-25*	+	+		+	*	+		+	*	*	*	Body image;Lifestyle
*BCNAS-32*		+	*	+		+		+		+		Logistics
*PCNQ V2*^*c*^		+	+	*		+		+			+	
*YYFcore03*	+	+	*		*	+	*	*	*	*		

* means the dimension is measured by one or more items within another dimension, or the item(s) were not assigned to a dimension by the instrument authors.

^a^These dimensions have been assigned by the reviewers. Definitions of the dimensions are in Online Resource 3.

^b^The dimension must include items that the respondent can indicate difficulties or a desire for help with. ^c^PCNQ V2 consists of two parts. This table focuses on the dimensions included in part two of the instrument.

### Clinimetric properties

#### Methodological quality

SPARC [19] and SCNAT-IP [33] were rated *adequate* for PROM development. The remaining instruments were rated *doubtful* (n = 11) [17, 20, 21, 28, 32, 35–37, 40, 41, 44] or *inadequate* (n = 11) [18, 22–27, 30, 31, 43, 46], due to a lack of pilot testing; failing to ask patients about comprehensibility; or failure to ensure or clarify, methodological detail, such as involving two researchers in the analysis. SPARC [19] and CNAT [28] were rated *adequate* for content validity. The remaining were *doubtful* (n = 11) [17, 20–22, 27, 32, 35, 36, 40, 41, 44] or *inadequate* (n = 11) [18, 23–26, 30, 31, 33, 37, 43, 46], primarily due to an insufficient sample of patients and professionals being asked about item relevance and comprehensiveness.

Twelve instruments were rated *very good* (n = 6) [18, 28, 32, 37, 41, 46] or *adequate* (n = 6) [31, 33, 35, 36, 40, 44] for structural validity. The remaining were *inadequate* for failing to conduct a factor analysis, or conducting analysis with an insufficient sample size. Twenty instruments were rated *very good* for internal consistency [17, 18, 20–23, 26–28, 30–33, 36, 37, 40, 41, 43, 44, 46]. The remaining were *doubtful* (n = 2) [19, 25] or *inadequate* (n = 2) [24, 35] for not being clear about, or measuring, internal consistency. For reliability, CSS-25 [41] was rated *very good*, and six others, *adequate* [17, 18, 27, 37, 44, 46]. One instrument [26] was rated *doubtful* because systematic change had occurred between assessment time points, while the remaining were *inadequate* for failing to test patients at different time points.

PCNA-EAV [26] and NEQ [37] were rated *adequate* for measurement error. The remaining were rated *inadequate* for not calculating the standard error of measurement, smallest detectable change, limits of agreement, or the percentage of agreement. Seven instruments were rated *very good* for hypotheses testing [27, 28, 32, 33, 40, 41, 43]. One instrument [26] was *doubtful* due to insufficient information on the measurement properties of comparator instruments. The remaining instruments were rated *inadequate* for either no information on the measurement properties of comparator instruments or no assessment of known groups validity. None of the instruments were tested for responsiveness (*Table* *4**, Online Resource* *11*).

**Table 4:** Methodological and quality assessment of each instrument

**Instrument**	*PROM development*	**Validity**					**Reliability**^**a**^					
		*Content validity*	*Structural validity*		*Hypotheses testing*		*Internal consistency*		*Reliability*		*Measurement error*	
	*M*	*M*	*M*	*Q*	*M*	*Q*	*M*	*Q*	*M*	*Q*	*M*	*Q*
*PNAP*	Inadequate	Inadequate	Very good	+	Inadequate	+	Very good	?	Adequate	?	Inadequate	?
*SPARC*	Adequate	Adequate	Inadequate	?	Inadequate	?	Doubtful	?	Inadequate	?	Inadequate	?
*NA-ACP*	Doubtful	Doubtful	Inadequate	?	Inadequate	?	Very good	+	Adequate	+	Inadequate	?
*NA-ALCP*	Doubtful	Doubtful	Inadequate	?	Inadequate	+	Very good	?	Inadequate	?	Inadequate	?
*PNPC*	Doubtful	Doubtful	Inadequate	?	Inadequate	+	Very good	?	Inadequate	?	Inadequate	?
*PNPC-sv*	Inadequate	Doubtful	Inadequate	?	Inadequate	+	Very good	?	Inadequate	?	Inadequate	?
*SPEED*	Inadequate	Inadequate	Inadequate	?	Inadequate	?	Very good	+	Inadequate	?	Inadequate	?
*3LNQ*	Inadequate	Inadequate	Inadequate	?	Inadequate	?	Inadequate	?	Inadequate	?	Inadequate	?
*Ndiok*	Inadequate	Inadequate	Inadequate	?	Inadequate	?	Doubtful	?	Inadequate	?	Inadequate	?
*PCNA-EAV*	Inadequate	Inadequate	Inadequate	?	Doubtful	+	Very good	?	Doubtful	?	Adequate	?
*CaNDI*	Inadequate	Doubtful	Inadequate	?	Very good	+	Very good	?	Adequate	+	Inadequate	?
*CNAT*	Doubtful	Adequate	Very good	+	Very good	+	Very good	+	Inadequate	?	Inadequate	?
*PNI*	Inadequate	Inadequate	Inadequate	?	Inadequate	?	Very good	?	Inadequate	?	Inadequate	?
*eHNA*	Inadequate	Inadequate	Adequate	?	Inadequate	?	Very good	?	Inadequate	?	Inadequate	?
*SCNS-SF34*	Doubtful	Doubtful	Very good	?	Very good	+	Very good	+	Inadequate	?	Inadequate	?
*SCNAT-IP*	Adequate	Inadequate	Adequate	?	Very good	+	Very good	+	Inadequate	?	Inadequate	?
*SCNS-ST9*	Doubtful	Doubtful	Adequate	?	Inadequate	?	Inadequate	?	Inadequate	?	Inadequate	?
*SCNS-LF59*	Doubtful	Doubtful	Adequate	?	Inadequate	?	Very good	+	Inadequate	?	Inadequate	?
*NEQ*	Doubtful	Inadequate	Very good	+	Inadequate	?	Very good	?	Adequate	?	Adequate	?
*CNQ-sf*	Doubtful	Doubtful	Adequate	?	Very good	+	Very good	+	Inadequate	?	Inadequate	?
*CSS-25*	Doubtful	Doubtful	Very good	+	Very good	+	Very good	?	Very good	+	Inadequate	?
*BCNAS-32*	Inadequate	Inadequate	Inadequate	?	Very good	+	Very good	+	Inadequate	?	Inadequate	?
*PCNQ V2*	Doubtful	Doubtful	Adequate	?	Inadequate	+	Very good	+	Adequate	+	Inadequate	?
*YYFcore03*	Inadequate	Inadequate	Very good	+	Inadequate	?	Very good	?	Adequate	+	Inadequate	?

^a^None of the instruments assessed responsiveness, so this was not reported.

M = Assessment of methodological quality: “Very good”, “Adequate”, “Doubtful”, “Inadequate”.

Q = Quality criteria for measurement properties: + = Sufficient, ? = Indeterminate, - = Insufficient.

#### GRADE levels of evidence

NA-ACP [17] and NA-ALCP [20] were the only instruments with *high* evidence for any clinimetric property, in both instances internal consistency (*Table* *5**, Online Resource* *12*). CSS-25 [41] appeared strongest overall, with *moderate* evidence for four properties. CNAT [28] and SCNS-SF34 [32] had *moderate* evidence for three properties. Eight instruments had at least *moderate* evidence for two properties [17, 18, 27, 33, 37, 40, 43, 46]. Eight instruments had at least *moderate* evidence for only one property [20–22, 26, 30, 31, 36, 44], all being internal consistency. The remaining five instruments had *low* or *very low* evidence across all seven clinimetric properties [19, 23–25, 35]. With the exception of internal consistency (for which two and 17 instruments had *high* and *moderate* evidence, respectively), *very low* evidence was common across all clinimetric properties.

**Table 5:** Levels of evidence (GRADE) for each instrument across clinimetric properties

**Instrument**	*PROM Development*	*Content Validity*	*Structural Validity*	*Hypotheses testing*	*Internal consistency*	*Reliability*	*Measurement error*
*PNAP*	-	-	††	-	††	†	-
*SPARC*	†	†	-	-	-	-	-
*NA-ACP*	†	†	-	-	†††	††	-
*NA-ALCP*	†	†	-	-	†††	-	-
*PNPC*	-	-	-	-	††	-	-
*PNPC-sv*	-	-	-	-	††	-	-
*SPEED*	-	-	-	-	†	-	-
*3LNQ*	-	-	-	-	-	-	-
*Ndiok*	-	-	-	-	†	-	-
*PCNA-EAV*	-	-	-	-	††	-	†
*CaNDI*	-	-	-	††	††	†	-
*CNAT*	-	†	††	††	††	-	-
*PNI*	-	-	-	-	††	-	-
*eHNA*	-	-	†	-	††	-	-
*SCNS-SF34*	-	-	††	††	††	-	-
*SCNAT-IP*	†	-	†	††	††	-	-
*SCNS-ST9*	-	-	†	-	-	-	-
*SCNS-LF59*	-	-	†	-	††	-	-
*NEQ*	-	-	††	-	††	†	†
*CNQ-sf*	-	-	†	††	††	-	-
*CSS-25*	-	-	††	††	††	††	-
*BCNAS-32*	-	-	-	††	††	-	-
*PCNQ V2*	-	-	†	-	††	†	-
*YYFcore03*	-	-	††	-	††	†	-

††† = High, †† = Moderate, † = Low, - = Very low

#### Quality of clinimetric properties

Across all properties, no instrument received an *insufficient* rating (*Table* *4**, Online Resource* *13*). Five instruments had *sufficient* structural validity [18, 28, 37, 41, 46]. The remaining instruments were *indeterminate*, primarily due to a lack of confirmatory factor analysis (CFA) or use of item response theory (IRT). eHNA [31] and SCNS-SF34 [32] conducted IRT and CFA, respectively, but failed to report the information required for a *sufficient* rating. Nine instruments had *sufficient* internal consistency [17, 23, 28, 32, 33, 36, 41, 43, 44]. The remaining were *indeterminate*, due to not having at least low evidence for sufficient structural validity; not reporting Cronbach’s alpha; or Cronbach’s alpha ranged below and above 0.70 across all unidimensional scales. Five instruments had *sufficient* reliability [17, 27, 41, 44, 46]; as COSMIN does not specify that the intraclass correlation coefficients (ICC) need to be above 0.70 in each dimension, studies were rated *sufficient* if at least one ICC was >0.70. The remaining were rated *indeterminate* for not reporting ICC or weighted Kappa. All 24 instruments had *indeterminate* measurement error [17–28, 30–33, 35–37, 40, 41, 43, 44, 46] for failing to define a minimal important change. Thirteen instruments had *sufficient* hypotheses testing [18, 20–22, 26–28, 32, 33, 40, 41, 43, 44], as they reported results in accordance with their hypotheses, while the remaining were *indeterminate* for failing to define a hypothesis.

#### Feasibility

Twelve instruments reported varying levels of patient comprehensibility (*Online Resource* *14*) [17, 19, 20, 24, 26, 27, 32, 33, 35–37, 44], while no instrument reported clinician comprehensibility. Only NA-ACP [17] and CaNDI [27] reported patients required mental and physical ability level, both considered by their authors to be understandable by > 90% of people aged 25–64 years.

Four instruments reported how to standardise scores, all using the same formula [32, 33, 35, 43]. Copyright was reported in five instruments [26, 31, 32, 35, 36]. Instrument access was available: within the paper (n = 11 instruments) [23, 25, 28, 30, 35–37, 40, 43, 44, 46]; as an appendix (n = 6) [20–22, 24, 26, 27]; to download online (n = 3) [19, 32, 41]; through another route (n = 3) [17, 31, 33]; and for PNAP [18] access was not reported.

Ten instruments were free to access [19, 31–33, 35–37, 40, 41, 44], while the remaining did not report cost of access. Five instruments were available in more than one format [27, 32, 33, 35, 36]. All except PCNA-EAV [26] (not reported) were stated to be available for use in a clinical setting. Sixteen instruments were also stated to be suitable for use in a research setting [17–22, 24, 25, 27, 28, 32, 35–37, 40, 41]. CSS-25 [41] reported additional usability in a community setting. No instrument reported requiring regulatory agency approval for use.

#### Interpretability

Through frequencies, mean and standard deviation, median, or range, 16 instruments reported the distribution of scores in the study population (*Online Resource* *15*) [21, 22, 24–28, 30–33, 35, 37, 40, 41, 43]. Nine instruments reported a percentage of missing items, either through the percentage missing for each individual item or percentage of the sample that missed ≥ 1 item [19, 20, 26, 27, 33, 37, 41, 43, 44]. Floor and ceiling effects were not applicable to the four instruments that included only dichotomous response options [21, 22, 25, 37]. Four instruments reported that such effects were either not observed or had been addressed through item reduction [19, 27, 32, 33]. CNAT [28] reported considerable floor effect and little ceiling effect. Eight instruments presented scores available for relevant subgroups, such as gender, age, treatment, and cancer type [26–28, 30–32, 37, 40]. No instrument reported a minimal important change/difference or provided information on response shift.

## Discussion

### Summary of main findings

This systematic review aimed to identify available unmet need instruments targeted at, or applicable to, people with advanced cancer. Overall, we identified 24 instruments. These were predominantly developed for all stages of cancer, with only eight specifically focused on advanced cancer [17, 20–26]. Hence, most authors did not specifically report how these instruments perform in advanced cancer populations. This study extends the work of Tian et al. who evaluated the psychometric properties of needs assessment tools in cancer [12]. Though our focus on advanced cancer was more specific than the work of Tian et al., we still included 11 additional instruments [18, 22, 25, 26, 30, 31, 36, 41, 43, 44, 46].

### Instrument development

Ten instruments incorporated patient discussions in their development, indicating consideration of the target population’s perspective. Of these, four also included health professional discussions or an expert panel to acquire both perspectives. However, according to the COSMIN criteria, the methodological quality of the included instruments was generally poor; indeed, SPARC [19], NA-ACP [17], and NA-ALCP [20] were the only instruments without very low evidence for both PROM development and content validity. Particularly, following development work, it was often unclear what constituted the final version of the instrument. This made aspects of our appraisal of the instruments challenging and would potentially have implications for others who might wish to use these instruments in research or practice.

### Instrument content

For consistency, we mapped the questions in the instruments to pre-defined dimensions (rather than rely on authors’ self-reported dimensions). There was substantial heterogeneity in the number of dimensions of unmet need assessed by each instrument. Twenty instruments assessed ≥ 7 dimensions, with PNPC [21] and PNI [30] assessing all 11 dimensions, while SPEED [23] (5 dimensions), SCNS-ST9 [32], BCNAS-32 [43], and PCNQ V2 [44] (6 dimensions) assessed the lowest number of dimensions. Hence, some instruments do not offer patients the opportunity to indicate difficulties, or a desire for help with, at least five dimensions of unmet need, potentially providing an incomplete picture of unmet needs at the individual or population level.

### Instrument quality

Despite the number of available instruments, methodological quality was variable. Only CSS-25 [41] (four properties), CNAT [28], and SCNS-SF34 [32] (three properties) had at least moderate evidence for three or more clinimetric properties. Though these three appear more clinimetrically robust than other instruments, they were developed for the whole cancer trajectory (rather than advanced cancer patients). Thus, it is unclear whether they are robust specifically for assessing unmet needs in advanced cancer. Of note, Moghaddam et al. argued, in the context of a systematic review of unmet needs in those with advanced cancer, that SCNS-SF34 neglects some dimensions of unmet need [8]. Since our search was completed in January 2021, the development of CancerSupportSource-15+ (CSS-15+), a shortened version of CSS-25, has been published [47]. The authors of that paper state that CSS-15+ is a brief, valid, and reliable multidimensional instrument that has strong correlation with CSS-25. As CSS-25 [41] was identified, clinimetrically, as the strongest instrument in the present review, CSS-15+ may warrant closer consideration.

### Selecting an instrument for use

Our particular interest was to identify instruments which may be used to assess unmet needs in patients with advanced cancer. Recommendations for which instrument to use may be informed by robust development, comprehensive content, or strong methodological quality, as outlined above. However, no particular instrument stands out in all of these aspects. Thus, recommendations may also be informed by instrument burden and ease of administration. Instrument length, completion time, and availability in different formats are arguably particularly important considerations for advanced cancer patients, who may, for example, have a significant symptom burden. Accordingly, eHNA [31], SCNS-ST9 [35], and NEQ [37] have favourable characteristics, though are variable in their content and quality.

A clinical setting may have the capacity or desire to deliver services and supports to address patient needs. All instruments in our review can be used in a clinical setting, so when used, have the potential to inform the development of a future care plan. However, many instruments can also be used in a research setting, and some care may be needed here. A needs assessment may raise the awareness or expectations of the patient. As Ahmed et al. have noted, if help is not going to be offered once a need has been identified, it could be counterproductive [48].

With the growth in availability and efficacy of the new precision and biological therapies [4] for those with advanced cancer, it is important to understand how – and if – instruments perform in capturing the needs of those treated by these new treatments. However, as none of the instruments states development or validation in a such population, future research should explore this, especially given the unique adverse effect profiles of these treatments.

### Reporting instrument development and validation

When using COSMIN to assess clinimetrics, it should be noted that we are not judging that something has not been done in a study; rather, we have made a judgement on what has been *reported*. Many development studies were published before the first iterations of the COSMIN checklist, and this may account, in part, for the low assessed quality. What has been reported for an instrument may be driven by what is deemed important by the authors and/or the word limit afforded in a medical journal. This may be insufficient to report the detail necessary for full COSMIN appraisal. In particular, methodological detail of development stages was often reported only very briefly. We would suggest that, in future, when reporting instrument development and validation, authors make use of supplementary material to provide additional methodological detail.

In 2019, a COSMIN study design checklist was established [49]. This allows authors to clarify the necessary detail for each stage of instrument development and validation. It would be helpful for scientific journals to require relevant studies be reported according to this checklist or that authors complete this checklist at submission and make it available.

### Strengths and limitations

The present review benefitted from an extensive search, including consideration of palliative care literature. This allowed us to identify instruments appropriate to those in palliative care (i.e. PNAP and SPARC). Still, this review is not without limitations. Though thorough in our review process by searching several databases and handsearching reference lists and citations, we only included papers and instruments available in English. Thus, the possibility cannot be excluded that we have missed a relevant study or instrument not published in English. While we signpost to available language validations, we only assessed the clinimetric properties of the original English versions of each instrument. If assessed, these may have influenced the GRADE summary of evidence. Although authors were contacted, complete development work for eHNA and CNQ-sf was not available. Therefore, it was not possible to accurately assess certain clinimetric properties.

One of the challenges in this review was what precisely constitutes an unmet need. We took the view that this is something that a patient experiences as a problem *and* which they would like help or support with. This meant we excluded instruments that simply measured problems or symptoms and did not allow patients to infer a need for help. One could argue that having a significant problem equates to an implicit need, and the authors for some of the excluded instruments may consider them measures of unmet needs or needs assessment tools. Indeed, there are examples in the literature where scores above a specified cut-off on functioning or symptom scales within a validated instrument are taken to infer an unmet need [50]. This highlights a lack of clarity for when a need is considered to be unmet and how such an unmet need is identified or measured.

Equally, there are challenges around defining ‘advanced’ cancer. For this work, advanced cancer was operationalised in terms of disease stage (stage IV) and ‘status’ (metastatic), also acknowledging any cancers that were considered incurable or people who were undergoing palliative care. It is possible to define advanced cancer in other ways, such as likely prognosis, chance of disease eradication, or patient remaining life expectancy. Study populations tend not to be described in these terms, and these concepts are, arguably, harder to quantify or categorise than stage or metastatic status. Both ‘unmet need’ and ‘advanced cancer’ are key concepts in survivorship. Consensus definitions of these would be valuable.

## Conclusion

We identified 24 instruments to measure unmet needs in people with advanced cancer. There is extensive heterogeneity in their development, content, and methodological quality. Moreover, the majority were not developed, or validated, with specific consideration of advanced cancer. The evolving management of advanced cancer, including the explosion in availability of precision and biological therapies, means it is important to consider whether existing instruments adequately capture the unmet needs of this population.

## Supplementary Information

ESM 1 (PDF 562 kb)

ESM 2 (XLSX 44 kb)

ESM 3 (PDF 114 kb)

## Data Availability

Not applicable
