# Supplementary material for: Assessing unmet needs in advanced cancer patients: a systematic review of the development, content, and quality of available instruments
Source: J Cancer Surviv. 2021 Aug 6;16(5):960–75. doi: 10.1007/s11764-021-01088-6 (PMC9489568; doi:10.1007/s11764-021-01088-6)
Supplement: Supplementary file 1 — (PDF 562 kb) [file 11764_2021_1088_MOESM1_ESM.pdf]

Assessing unmet needs in advanced cancer patients: a systematic review of the development, content, and quality of available instruments

Ben Rimmer[1*], Lisa Crowe[1], Adam Todd[1,2+], Linda Sharp[1+]

Author affiliations:

1. *Population Health Sciences Institute, Newcastle University, Newcastle University Centre for Cancer, Newcastle, England*
2. *School of Pharmacy, Newcastle University, Newcastle, England*

**Corresponding author: Ben Rimmer, Population Health Sciences Institute, Newcastle University, Sir James Spence Institute, Royal Victoria Infirmary, Newcastle upon Tyne, NE1 4LP.*
Email: ben.rimmer@newcastle.ac.uk; Phone: 0044 (0)7704 300 509

+Joint senior authors

Contents

Online Resource 1: Search strategy.......2
Online Resource 2: Database search example.......3
Online Resource 3: Dimension definitions.......4
Online Resource 4: Definitions and COSMIN quality criteria for good measurement properties.......6
Online Resource 5: GRADE levels of evidence.......8
Online Resource 6: Instrument purpose, setting and characteristics of the validation study population.......9
Online Resource 7: Validation studies in languages other than English.......13
Online Resource 8: Other techniques used in instrument development.......15
Online Resource 9: References to support development quality appraisal.......16
Online Resource 10: Dimension development.......17
Online Resource 11: Overall methodological quality appraisal.......19
Online Resource 12: GRADE levels of evidence with reasons.......19
Online Resource 13: Quality appraisal with reasons.......19
Online Resource 14: Instrument feasibility.......20
Online Resource 15: Instrument interpretability.......25

Online Resource 1: Search strategy

Key area	Keywords
1. Cancer	cancer OR neoplasm/
2. Advanced Disease	advanced OR metastatic OR palliative OR chronic OR end of life OR late stage OR terminal
3. Needs	unmet need* OR need* assess* OR perceived need* OR support* care need* OR psycho* need* OR physical need* OR information* need* OR function* need*
4. Instrument	instrument* OR measure* OR scale* OR survey* OR questionnaire* OR tool* OR inventor* OR checklist*

Online Resource 2: Database search example

MEDLINE search (Ovid MEDLINE® 1946 to February Week 4 2020)

# ▲	Searches	Results
1	Neoplasms/	415111
2	cancer.mp.	1445236
3	1 or 2	1629056
4	advanced.mp.	348001
5	metastatic.mp.	188986
6	palliative.mp.	76504
7	chronic.mp.	1188586
8	end of life.mp.	19105
9	late stage.mp.	17948
10	terminal.mp.	397605
11	4 or 5 or 6 or 7 or 8 or 9 or 10	2110788
12	unmet need*.mp.	9913
13	need* assess*.mp.	33079
14	perceived need*.mp.	2615
15	support* care need*.mp.	555
16	psycho* need*.mp.	3041
17	physical need*.mp.	376
18	information* need*.mp.	6554
19	function* need*.mp.	1217
20	12 or 13 or 14 or 15 or 16 or 17 or 18 or 19	54039
21	instrument*.mp.	851230
22	measure*.mp.	2865427
23	scale*.mp.	683385
24	survey*.mp.	895181
25	questionnaire*.mp.	646244
26	tool*.mp.	570111
27	inventor*.mp.	91075
28	checklist*.mp.	32150
29	21 or 22 or 23 or 24 or 25 or 26 or 27 or 28	4997176
30	3 and 11 and 20 and 29	747

Online Resource 3: Dimension definitions

Dimension	Definition	Example items
Physical	Needs relating to a symptom of diagnosis or side effect of treatment.	• PCNA-EAV – Dealing with difficulty breathing<br>• SCNS-SF34 – Lack of energy/tiredness<br>• PNAP – Being pain free
Psychological	Needs relating to feelings and emotions surrounding the cancer journey.	• YYFcore03 – Feeling down or depressed<br>• NA-ACP – Dealing with anxiety or stress<br>• CNAT – Feelings of anger, irritability, or nervousness
Information	Needs for more information, knowledge, or understanding about all aspects of the cancer journey.	• Ndiok – I need more information about my diagnosis<br>• PNI – Information about medication and side effects<br>• SCNS-LF59 – To be given written information about the important aspects of your care
Social	Needs concerning relationships with friends, family, and partners.	• PNPC-SV – Problems in the relationship with life companion<br>• 3LNQ – Difficulties with family life and contact to friends<br>• CNQ-SF – Coping with changes in other people's attitudes and behaviour towards you
Activities of daily living	Needs relating to essential activities necessary for everyday functioning.	• PNAP – Being able to take care of your body (washing, dressing, food)<br>• eHNA – Preparing meals or drinks<br>• SPARC – Changes in your ability to carry out your usual household tasks such as cooking for yourself or cleaning the house
Health care	Needs relating to the individual's access to treatment and/or support services and their relationship with health professionals.	• SPEED – How much difficulty are you having getting medical care that fits with your goals?<br>• SCNAT-IP – Having access to professional counselling if you or your family and friends need it<br>• PCNQ V2 – My specialist(s) are very supportive
Spiritual	Needs that relate to finding meaning, purpose, and value in life, though not necessarily religious.	• SPARC – Religious or spiritual needs not being met<br>• NA-ACP – Clarifying your spiritual beliefs<br>• PNPC – Difficulties concerning the meaning of death
Sexual	Needs relating to an individual's intimate relationships and sexual function.	• 3LNQ – Difficulties with sex life<br>• SCNS-ST9 – Changes in sexual relationships<br>• CSS-25 – Intimacy, sexual function and/or fertility
Economic	Needs relating to an individual's finances and resources.	• NA-ALCP – Dealing with concerns about your financial situation<br>• NEQ – I need economic help<br>• CSS-25 – Health insurance or money worries
Autonomy	Needs relating to an individual's ability to do things for themselves.	• Ndiok – Frustrations because one can do less than before<br>• CaNDI – Concerns about being unable to take care of myself

Dimension	Definition	Example items
Role	Needs relating to an individual's responsibilities and the part they play at home and in society.	• PNPC-SV – Difficulties in continuing the usual activities<br>• PNPC – Difficulties in caring for children or babysitting<br>• CaNDI – Managing work, school, or home life<br>• eHNA – Work and education

Online Resource 4: Definitions and COSMIN quality criteria for good measurement properties

Measurement property			Definition[a]	Quality criteria for each property[b,c]
Validity	Content validity		The degree to which the content of a PROM is an adequate reflection of the construct to be measured.	Quality not assessed.
	Construct validity	Structural validity	The degree to which the scores of a PROM are an adequate reflection of the dimensionality of the construct to be measured.	+: CTT: CFA: CFI or TLI or comparable measure >0.95 OR RMSEA <0.08<br>IRT/Rasch: No violation of unidimensionality: CFI or TLI or comparable measure >0.95 OR RMSEA <0.06 OR SRMR <0.08<br>AND no violation of local independence: residual correlations among the items after controlling for the dominant factor <0.20 OR Q3's <0.37<br>AND no violation of monotonicity: adequate looking graphs OR item scalability >0.30<br>AND adequate model fit: IRT: χ2 >0.01<br>Rasch: infit and outfit mean squares ≥ 0.5 and ≤ 1.5 OR Z- standardized values > -2 and <2.<br>**?**: CTT: Not all information for '+' reported.<br>IRT/Rasch: Model fit not reported.<br>**-**: Criteria for '+' not met.
		Hypotheses testing	The degree to which the scores of the instrument are consistent with the theoretically derived hypotheses.	+: The result is in accordance with the hypothesis.<br>**?**: No hypothesis defined (by the review team)<br>**-**: The result is not in accordance with the hypothesis.
Reliability	Reliability		The proportion of the total variance in the measurements which is due to 'true' differences between patients.	+: ICC or weighted Kappa ≥ 0.70.<br>**?**: ICC or weighted Kappa not reported.<br>**-**: ICC or weighted Kappa <0.70.
	Internal consistency		The degree of the interrelatedness among the items.	+: At least low evidence for sufficient structural validity AND Cronbach's alpha(s) ≥ 0.70 for each unidimensional scale or subscale<br>**?**: Criteria for "At least low evidence for sufficient structural validity" not met<br>**-**: At least low evidence for sufficient structural validity AND Cronbach's alpha(s) < 0.70 for each unidimensional scale or subscale
	Measurement error		The systematic and random error of a patient's score that	+: SDC or LoA < MIC.<br>**?**: MIC not defined.

Measurement property	Definition[a]	Quality criteria for each property[b,c]
	is not attributed to true changes in the construct to be measured.	**-**: SDC or LoA > MIC.
Responsiveness	The ability of a PROM to detect change over time in the construct to be measured.	**+**: The result is in accordance with the hypothesis OR AUC ≥ 0.70.<br>**?**: No hypothesis defined (by the review team).<br>**-**: The result is not in accordance with the hypothesis7 OR AUC < 0.70.

[a]Definitions of the measurement properties are based on Mokkink et al. (2010).
[b]Quality criteria are based on Terwee et al. (2007) and Prinsen et al. (2016).
[c] “+” = sufficient, “?” = indeterminate, “-” = insufficient

AUC = area under the curve, CFA = confirmatory factor analysis, CFI = comparative fit index, CTT = classical test theory, DIF = differential item functioning, ICC = intraclass correlation coefficient, IRT = item response theory, LoA = limits of agreement, MIC = minimal important change, RMSEA: Root Mean Square Error of Approximation, SEM = Standard Error of Measurement, SDC = smallest detectable change, SRMR: Standardized Root Mean Residuals, TLI = Tucker-Lewis index

Online Resource 5: GRADE levels of evidence

<table>
<tr><th>Quality of evidence</th><th>Lower if:</th></tr>
<tr><td>High (†††)</td><td rowspan="4">Risk of bias
-1 Serious
-2 Very serious
-3 Extremely serious

Inconsistency
-1 Serious
-2 Very serious

Imprecision
-1 total n=50-100
-2 total n<50

Indirectness
-1 Serious
-2 Very serious</td></tr>
<tr><td>Moderate (††)</td></tr>
<tr><td>Low (†)</td></tr>
<tr><td>Very low (-)</td></tr>
</table>

n = sample size

Online Resource 6: Instrument purpose, setting and characteristics of the validation study population

Instrument	Instrument purpose	Study setting	Study population
PNAP	To identify the needs of patients receiving palliative care.	Czech Republic	Mixed palliative chronic disease and cancer patients (n = 349):<br>Cancer - n = 225 (64.5%);<br>Chronic disease - n = 124 (35.5%);<br><br>Mouth, pharynx, gastrointestinal tract - n = 54 (24%);<br>Respiratory - n = 37 (16%);<br>Breast, female reproductive organs - n = 43 (19%);<br>Male reproductive organs - n = 19 (8%);<br>Urinary bladder - n = 10 (4%);<br>Eye, central nervous system - n = 11 (5%);<br>Hematopoesis - n = 18 (8%);<br>Other cancers - n = 16 (7%)
SPARC	To facilitate the referral of patients with advanced illnesses (regardless of diagnosis) into specialist palliative care.	UK	Patients with an advanced illness (n = 100)
NA-ACP	To measure the perceived needs of patients with advanced, incurable cancer.	Australia	Mixed advanced cancer patients (n = 246):<br>Breast - n = 59 (24%);<br>Bowel/Colon - n = 47 (19%);<br>Lung - n = 26 (11%);<br>Lymph node - n = 25 (10%);<br>Ovary - n = 13 (5%);<br>Prostate - n = 9 (4%);<br>Mouth/throat - n = 8 (3%);<br>Other - n = 51 (21%);<br>Unreported - n = 8 (3%)
NA-ALCP	To assess the needs of patients with advanced, incurable lung cancer.	Australia	Advanced lung cancer patients (n = 108)
PNPC	To investigate problems experienced by patients and (unmet) needs for care.	Netherlands	Mixed palliative cancer patients (those with distant mets) (n = 64):<br>Breast - n = 29 (2 of whom also had cervical cancer) (46%);<br>Lung - n = 3 (4.7%);<br>Esophagus/stomach - n = 1 (1.6%);<br>Colon/ rectum - n = 11 (17.2%);<br>Ovary - n = 5 (7.8%);<br>Malignant melanoma - n = 4 (6.3%);<br>Cervix - n = 3 (2 of whom also had breast cancer) (4.7%);<br>Prostate - n = 2 (3.1%);<br>Neck/ head - n = 1 (1.6%);<br>Hematological/ lymph system - n = 1 (1.6%);<br>Primary liver cancer - n = 1 (1.6%);<br>Pancreas - n = 1 (1.6%);<br>Unknown - n = 4 (6%)
PNPC-sv	To measure all dimensions of palliative care, investigating patients' problems and (unmet) needs	Netherlands	Mixed patients with metastatic cancer (n = 94):<br>Breast - n = 37 (39%);<br>Colorectal - n = 14 (15%);<br>Lung - n = 8 (8%);<br>Prostate - n = 6 (6%);<br>Ovary - n = 5 (5%);

Instrument	Instrument purpose	Study setting	Study population
			Malignant melanoma - n = 4 (4%);<br>Unreported - n = 21 (23%)
SPEED	To screen for palliative care needs of patients with cancer presenting to the emergency department.	USA	Mixed cancer patients with metastatic disease (n = 49):<br>Breast - n = 8 (16%);<br>Colon - n = 7 (14%);<br>Lung - n = 7 (14%);<br>Lymphoma - n = 6 (11%);<br>Unreported - n = 21 (45%)
3LNQ	To measure palliative needs in three ways: problem intensity, problem burden, and felt need.	Denmark	Advanced cancer patients (n = 74):<br>Stage III - n = 38 (51%);<br>Stage IV - n = 30 (41%);<br>Unreported - n = 6 (8%)<br><br>Brain - n = 2 (3%);<br>Gastrointestinal - n = 24 (32%);<br>Gynecological - n = 10 (13%);<br>Head and neck - n = 5 (7%);<br>Breast - n = 17 (23%);<br>Lung - n = 2 (3%);<br>Hematological - n = 5 (7%);<br>Genitourinary - n = 6 (8%)<br>Other - n = 3 (4%)
Ndiok	To assess palliative care needs of patients with cancer from the perspective of the patients themselves.	Nigeria	Mixed cancer patients (n = 429):<br>Breast - n = 165 (38.5%);<br>Cervix - n = 44 (10.3%);<br>Prostate - n = 73 (17%);<br>Skin - n = 23 (5.4%);<br>Lung - n = 50 (11.6%);<br>Other - n = 69 (16.1%);<br>Unreported - n = 5 (1.1%)
PCNA-EAV	To measure the health care and support care needs of patients with advanced cancer.	Saudi Arabia	Mixed advanced cancer patients (n = 50):<br>Breast - n = 14 (28%);<br>Lung - n = 4 (8%);<br>Liver - n = 5 (10%);<br>Gastrointestinal - n = 3 (6%);<br>Genitourinary - n = 1 (2%);<br>Lymphoma - n = 9 (18%);<br>Colon - n = 9 (18%);<br>Other - n = 5 (10%)
CaNDI	To identify distressed patients in need of intervention.	USA	Mixed cancer patients (n = 100):<br>Stage I - n = 22 (22%);<br>Stage II - n = 19 (19%);<br>Stage III - n = 27 (27%);<br>Stage IV - n = 19 (19%);<br>Unreported - n = 13 (13%)<br><br>Breast - n = 30 (30%);<br>CLL - n = 10 (10%);<br>Colon/ rectal - n = 13 (13%);<br>Lung - n = 6 (6%);<br>Myeloma - n = 4 (4%);<br>Ovarian - n = 10 (10%);<br>Other - n = 17 (17%);<br>Multiple site - n = 6 (6%);<br>Missing - n = 4 (4%)
CNAT	To cover cancer patients' needs in a comprehensive way throughout all phases	Republic of Korea	Mixed cancer patients (n = 2661):<br>Stomach - n = 399 (15%);<br>Lung - n = 287 (10.8%);

Instrument	Instrument purpose	Study setting	Study population
	of the cancer experience, from diagnosis to recovery or palliative care		Colon/ rectum - n = 324 (12.2%);<br>Breast - n = 325 (12.2%);<br>Cervix - n = 98 (3.7%);<br>Other - n = 1008 (37.9%)
PNI	To identify the psychosocial needs of cancer patients, and the contributory factors to need.	UK	Mixed cancer patients (n = 402):<br>Breast - n = 183 (46%);<br>Colorectal - n = 121 (30%);<br>Lung - n = 62 (15%);<br>Lymphoma - n = 36 (9%)
eHNA	To help people living with cancer express all their needs and help those helping them better target support.	UK	Mixed cancer patients (n = 5421)
SCNS-SF34	To measure cancer patients' perceived needs across a range of domains.	Australia	Three samples of mixed cancer patients (n = 444, 444, 250):<br>Breast - n = 137 (31%), 139 (31%), 14 (6%);<br>Colorectal - n = 74 (17%), 70 (16%), 21 (8%);<br>Prostate - n = 39 (9%), 35 (8%), 82 (33%);<br>Lung - n = 31 (7%), 32 (7%), 23 (9%);<br>Other - n = 137 (31%), 142 (32%), 110 (44%)
SCNAT-IP	To measure indigenous cancer patients' perceived needs across a range of domains.	Australia	Mixed indigenous cancer patients (n = 29):<br>Gynecological - n = 9 (31%);<br>Lung - n = 7 (24%);<br>Breast - n = 5 (14%);<br>Blood - n = 3 (10%);<br>Bowel - n = 2 (7%);<br>Brain - n = 1 (3%);<br>Prostate - n = 1 (3%);<br>Thyroid - n = 1 (3%)
SCNS-ST9	A brief screening tool to assess the perceived needs of people with cancer.	Australia	Two samples of mixed cancer patients (n = 977 (development), 481 (validation)):<br>Current cancer site(s) reported in n = 1345):<br>Breast - n = 232 (25.6%), 117 (26.9%);<br>Bowel/colon/rectum - n = 99 (10.9%), 49 (11.3%);<br>Lung - n = 84 (9.3%), 35 (8%);<br>Prostate - n = 66 (7.3%), 40 (9.2%);<br>Melanoma/skin - n = 42 (4.6%), 14 (3.2%);<br>Cervical/ovarian - n = 33 (3.6%), 15 (3.4%);<br>Brain tumour - n = 28 (3.1%), 13 (3%);<br>Other - n = 323 (35.6%), 152 (34.9%)
SCNS-LF59	To assess the generic needs of patients with cancer.	Australia	Mixed cancer patients (n = 888):<br>Breast - n = 280 (32%);<br>Colon and rectum - n = 150 (17%) ;<br>Prostate - n = 80 (9%);<br>Lung - n = 67 (8%);<br>Skin/melanoma - n = 43 (5%);<br>Don't know - n = 14 (2%);<br>Other - n = 217 (24%)
NEQ	To evaluate needs expressed by (hospitalised) cancer patients.	Italy	Mixed cancer patients (n = 423):<br>Colon-rectum - n = 114 (27%);<br>Genitourinary - n = 114 (27%);<br>Breast - n = 93 (22%);<br>Not reported - n = 102 (24%)
CNQ-sf	To assess cancer patient's needs across several domains.	Australia	Mixed cancer patients (n = 450):<br>No evidence - n = 193 (43%);<br>Localised, asymptomatic - n = 59 (13%);<br>Localised, symptomatic - n = 68 (15%);<br>Metastatic, asymptomatic - n = 45 (10%);

Instrument	Instrument purpose	Study setting	Study population
			Metastatic, symptomatic - n = 85 (19%)<br><br>Lung - n = 132 (29%);<br>Head and neck - n = 95 (21%);<br>Gynaecological - n = 77 (17%);<br>Haematology/Lymphoma - n = 45 (10%) ;<br>Melanoma - n = 45 (10%);<br>Other - n = 54 (12%)
CSS-25	To screen for psychosocial distress among cancer patients.	USA	Mixed cancer patients (n = 251):<br>Stage 0 - n = 10 (4%);<br>Stage I - n = 58 (25%);<br>Stage II - n = 57 (24%);<br>Stage III - n = 44 (19%);<br>Stage IV - n = 39 (17%);<br>Do not know - n = 26 (11%)<br><br>Breast - n = 108 (46%);<br>Gynecologic - n = 20 (9%);<br>Blood - n = 17 (7%);<br>Colorectal - n = 14 (6%);<br>Lung - n = 9 (4%);<br>Prostate - n = 9 (4%);<br>Other - n = 43 (18%);<br>Multiple reported - n = 16 (7%)
BCNAS-32	To provide a comprehensive assessment of the multi-dimensional impact of bladder cancer on patients' outcomes and their current needs for support.	USA	Bladder cancer patients (n = 159):<br>Stage 0 - n = 29 (18%);<br>Stage I - n = 59 (37%);<br>Stage II - n = 40 (25%);<br>Stage III - n = 13 (8%);<br>Stage IV - n = 10 (6%);<br>Do not know - n = 5 (3%)
PCNQ V2	To measure the perceived needs of men diagnosed with prostate cancer.	Australia	Prostate cancer patients (n = 300):<br>Localised - n = 172 (57%);<br>Advanced - n = 50 (17%);<br>Do not know - n = 78 (26%)
YYFcore03	To screen for biopsychosocial distress of newly diagnosed cancer patients in the outpatient setting.	USA	Mixed cancer patients (n = 608):<br>Breast - n = 262 (43%);<br>Genitourinary - n = 77 (13%);<br>Other organs - n = 80 (13%);<br>Prostate/testes - n = 59 (10%);<br>Benign/non-neoplasm - n = 130 (21%)

Online Resource 7: Validation studies in languages other than English

Instrument[a]	Language	Reference
SPARC	Polish	Leppert W, Majkowicz M, Ahmedzai SH. The adaptation of the Sheffield Profile for Assessment and Referral for Care (SPARC) to the Polish clinical setting for needs assessment of advanced cancer patients. J Pain Symptom Manage. 2012 Dec 1;44(6):916-22. https://doi.org/10.1016/j.jpainsymman.2011.12.286
PNPC-sv	Chinese	Wang T, Molassiotis A, Chung BP, Tan JY. Psychometric assessment of the Chinese version of the Problems and Needs in Palliative Care questionnaire-short version in advanced cancer patients. BMC Palliat Care. 2019 Dec;18(1):1-2. https://doi.org/10.1186/s12904-019-0450-5
CaNDI	Turkish	Beyhun NE, Can G, Tiryaki A, Karakullukcu S, Bulut B, Yesilbas S, Kavgaci H, Topbas M. Validity and Reliability of the Turkish Version of Needs Based Biopsychosocial Distress Instrument for Cancer Patients (CANDI). Iran Red Crescent Med J. 2016 Jun;18(6). https://doi.org/10.5812/ircmj.27352
CNAT	Chinese	Zhang YP, Wei HH, Zhao XS, Zhang Y, Xu YQ, Porr C. Cross-Cultural Adaptation and Validation of the Comprehensive Needs Assessment Tool (CNAT) for Chinese Cancer Patients. Eur J Pers Cent Healthc. 2016 Jun 24;4(1):241-9. http://dx.doi.org/10.5750/ejpch.v4i1.1078
SCNS-SF34	Dutch	Li WW, Lam WW, Shun SC, Lai YH, Law WL, Poon J, Fielding R. Psychometric assessment of the Chinese version of the Supportive Care Needs Survey short-form (SCNS-SF34-C) among Hong Kong and Taiwanese Chinese colorectal cancer patients. PLoS one. 2013 Oct 11;8(10):e75755. https://doi.org/10.1371/journal.pone.0075755
	French	Bredart A, Kop JL, Griesser AC, Zaman K, Panes-Ruedin B, Jeanneret W, DELALOYE JF, Zimmers S, Jacob A, Berthet V, Fiszer C. Validation of the 34-item Supportive Care Needs Survey and 8-item breast module French versions (SCNS-SF34-Fr and SCNS-BR8-Fr) in breast cancer patients. Eur J Cancer Care. 2012 Jul;21(4):450-9. https://doi.org/10.1111/j.1365-2354.2012.01356.x
	German	Lehmann C, Koch U, Mehnert A. Psychometric properties of the German version of the short-form supportive care needs survey questionnaire (SCNS-SF34-G). Support Care Cancer. 2012 Oct;20(10):2415-24. https://doi.org/10.1007/s00520-011-1351-1
	Italian	Zeneli A, Fabbri E, Donati E, Tierney G, Pasa S, Berardi MA, Maltoni M. Translation of supportive care needs survey short form 34 (SCNS-SF34) into Italian and cultural validation study. Support Care Cancer. 2016 Feb 1;24(2):843-8. https://doi.org/10.1007/s00520-015-2852-0
	Japanese	Okuyama T, Akechi T, Yamashita H, Toyama T, Endo C, Sagawa R, Uchida M, Furukawa TA. Reliability and validity of the Japanese version of the Short-form Supportive Care Needs Survey Questionnaire (SCNS-SF34-J). Psychooncology. 2009 Sep;18(9):1003-10. https://doi.org/10.1002/pon.1482
	Mandarin and Cantonese	Au A, Lam WW, Kwong A, Suen D, Tsang J, Yeo W, Suen J, Ho WM, Yau TK, Soong I, Wong KY. Validation of the Chinese version of the short-form Supportive Care Needs Survey Questionnaire (SCNS-SF34-C). Psychooncology. 2011 Dec;20(12):1292-300. https://doi.org/10.1002/pon.1851
	Mandarin	Han Y, Zhou Y, Wang J, Zhao Q, Qin H, Fan Y, Song Y, Boyes A, Cui S. Psychometric testing of the Mandarin version of the 34-item Short-Form Supportive Care Needs Survey in patients with cancer in mainland China. Support Care Cancer. 2017 Nov;25(11):3329-38. https://doi.org/10.1007/s00520-017-3750-4

**Instrument**[a]	**Language**	**Reference**
	Mexican	Doubova SV, Aguirre-Hernandez R, Gutiérrez-de la Barrera M, Infante-Castañeda C, Pérez-Cuevas R. Supportive care needs of Mexican adult cancer patients: validation of the Mexican version of the Short-Form Supportive Care Needs Questionnaire (SCNS-SFM). Support Care Cancer. 2015 Sep;23(9):2711-9. https://doi.org/10.1007/s00520-015-2634-8
	Nepali	Dhakal K, Wang P, Mboineki JF, Getu MA, Chen C, Boyes A, Sharma C, Ghimire BR, Adhikari A, Adhikari B. Translation of Supportive Care Needs Survey Short Form 34 (SCNS-SF34) into Nepali Language with Cultural Validation. Pre-print. https://doi.org/10.21203/rs.3.rs-213979/v1
	Traditional Chinese	Li WW, Lam WW, Shun SC, Lai YH, Law WL, Poon J, Fielding R. Psychometric assessment of the Chinese version of the Supportive Care Needs Survey short-form (SCNS-SF34-C) among Hong Kong and Taiwanese Chinese colorectal cancer patients. PLoS one. 2013 Oct 11;8(10):e75755. https://doi.org/10.1371/journal.pone.0075755
	Turkish	Özbayır T, Geçkil ÖS, Aslan A. An adaptation of the short-form supportive care needs survey questionnaire (SCNS-SF 34) into Turkish. Eur J Breast Health. 2017 Oct;13(4):183. https://dx.doi.org/10.5152%2Ftjbh.2017.3266
SCNS-ST9	Chinese	Chan-Chuan HU, Kai-Lin YO, Li-Yun TS, Yuan-Yuan FA, Jhang SY, Pei-Jen LO, Cheng-Ping WA, Jenq-Yuh KO, Yun-Hsiang LE, Yeur-Hur LA. Validation of the Supportive Care Needs Survey Screening Tool Chinese Version for Patients With Head and Neck Cancer in Taiwan. J Nurs Res. 2019 Dec 1;27(6):e50. https://doi.org/10.1097/jnr.0000000000000360
NEQ	Greek	Konstantinidis TI, Samonis G, Sarafis P, Philalithis A. Assessment of needs of hospitalized cancer patients with advanced cancer. Glob J Health Sci. 2017;9:184-4. http://dx.doi.org/10.5539/gjhs.v9n6p184
CNQ-sf	Arabic	Sharour LA. Translation and Validation of the Arabic Version of the Cancer Needs Questionnaire-Short Form. Asia Pac J Oncol Nurs. 2021 Jan;8(1):74. https://dx.doi.org/10.4103%2Fapjon.apjon_33_20
	Chinese	Chen SC, Lai YH, Cheng SY, Liao CT, Chang JT. Psychometric testing of the Chinese-version cancer needs questionnaire short form head and neck cancer-specific version in oral cavity cancer patients. Support Care Cancer. 2011 May;19(5):647-56. https://doi.org/10.1007/s00520-010-0877-y

[a]Though the review included 24 instruments, this table only includes those with an additional language validation.

Online Resource 8: Other techniques used in instrument development

Instrument	Item generation	Item reduction
SPARC		Cognitive interviews with professionals; item analysis
NA-ALCP		Test-retest reliability
PNPC	Caregiver interviews	
PCNA-EAV	Author knowledge	
CaNDI		Health professional focus group
SCNS-SF34		Floor/ceiling effects; Item-total correlations; Author review (for “clinical relevance”)
SCNAT-IP	Key informant focus groups	Key informant focus groups
SCNS-ST9		Sensitivity and specificity; Item variation explained; ROC curve analysis; Positive and negative predictive value
NEQ		Problematic items eliminated after initial field-testing; Test-retest reliability
YYFcore03	Patient data	

Online Resource 9: References to support development quality appraisal

Instrument	Reference
SPARC	Bestall JC, Ahmed N, Ahmedzai SH, Payne SA, Noble B, Clark D. Access and referral to specialist palliative care: patients' and professionals' experiences. Int J Palliat Nurs. 2004 Aug;10(8):381-9. https://doi.org/10.12968/ijpn.2004.10.8.15874
PNPC	Osse BH, Vernooij-Dassen MJ, Schadé E, de Vree B, van den Muijsenbergh ME, Grol RP. Problems to discuss with cancer patients in palliative care: a comprehensive approach. Patient Educ Couns. 2002 Jul 1;47(3):195-204. https://doi.org/10.1016/S0738-3991(02)00019-8
CaNDI	Clark K, Bardwell WA, Arsenault T, DeTeresa R, Loscalzo M. Implementing touch-screen technology to enhance recognition of distress. Psychooncology. 2009 Aug;18(8):822-30. https://doi.org/10.1002/pon.1509
SCNS[a]	Foot G, Sanson-Fisher R. Measuring the unmet needs of people living with cancer. Cancer Forum 1995 (Vol. 19, No. 2, pp. 131-135).
BCNAS-32	Mohamed NE, Herrera PC, Hudson S, Revenson TA, Lee CT, Quale DZ, Zarcadoolas C, Hall SJ, Diefenbach MA. Muscle invasive bladder cancer: examining survivor burden and unmet needs. J Urol. 2014 Jan 1;191(1):48-53. https://doi.org/10.1016/j.juro.2013.07.062
YYFcore03	Loscalzo MJ, Clark KL. Problem-related distress in cancer patients drives requests for help: a prospective study. Oncology. 2007 Aug 1;21(9):1133.

[a]References listed for SCNS are applicable to the LF59, SF34, and ST9.

Online Resource 10: Dimension development

Instrument	Dimension development	Dimensions (as described by authors of the validation study(ies) included in the review)
PNAP	Confirmatory factor analysis; Health professional focus group	Physical symptoms; Social area; Respect and support from health professionals; Meaning of life and reconciliation; Autonomy; Chance to share emotions; Religious needs
SPARC	Literature; Author grouped	Communication and information issues; Physical symptoms; Psychological issues; Religious and spiritual issues; Independence and activity; Family and social issues; Treatment issues
NA-ACP	Principal component analysis; Literature	Medical communication/information; Psychological/Emotional; Daily living; Financial; Symptom; Spiritual; Social
NA-ALCP	Existing instrument	Medical communication; Psychological/emotional; Daily living; Financial; Symptom; Spiritual/existential; Social
PNPC	Literature; Patient interviews	Activities of daily living; Physical symptoms; Role activities; Financial and administrative issues; Social issues; Psychological issues; Spiritual issues; Autonomy; Information needs; Problems in consultations; Overriding problems in quality of care; Concerning the GP; Concerning the specialist
PNPC-sv	Existing instrument	Daily activities; Physical symptoms; Autonomy; Social issues; Psychological issues; Spiritual issues; Financial issues; Need of information
SPEED	Literature; Expert panel	Physical; Spiritual; Social; Therapeutic; Psychological
3LNQ	Literature; Existing instrument	Physical function; Role function; Depression; Worry; Concentration; Social function; Fatigue; Nausea; Pain; Dyspnea; Reduced appetite; Sexuality; Feeling as a burden; Loneliness
Ndiok	Conceptual needs model	Information needs; Spiritual issues; Psychological issues; Activities of daily living; Physical symptoms; Financial issues; Social issues; Self-sufficiency; Problems in consultations; Problems in quality of care; Concerning the nurses
PCNA-EAV	Literature; Review of existing instruments	Physical/functional; Social; Psychological/emotional; Information; Communication; Helpful resources; Financial; Religious/spiritual; Priority of need; Preference for care
CaNDI	Biopsychosocial model	Depression; Anxiety; Emotion; Social; Health care; Practical; Physical
CNAT	Exploratory factor analysis	Health care staff; Psychological problems; Physical symptoms; Information; Social/religious/spiritual support; Practical support; Hospital facilities and services;
PNI	Author grouped	Health professional; Information; Support network; Identity; Emotional and spiritual; Practical; Practical: child
eHNA	Exploratory factor analysis; Principal component analysis	Emotional/spiritual; Oral effects; Activities of daily living; Caring/relationships; Surgery; Physiological effects; Toilet/pain; Gut; Treatment outcomes; Practical; Sexuality; Weight
SCNS-SF34	Exploratory factor analysis; Confirmatory factor analysis; Existing instrument	Psychological; Health systems and information; Patient care and support; Physical and daily living; Sexuality
SCNAT-IP	Existing instrument	Physical and Psychological; Health care; Practical and Cultural; Information and Communication

Instrument	Dimension development	Dimensions (as described by authors of the validation study(ies) included in the review)
SCNS-ST9	Existing instrument	Psychological; Health systems and information; Patient care and support; Physical and daily living; Sexuality
SCNS-LF59	Principal component analysis; Existing instrument	Psychological; Health systems and information; Patient care and support; Physical and daily living; Sexuality
NEQ	Exploratory factor analysis; Confirmatory factor analysis	Tamburini: Information (diagnosis & prognosis); Information (exams & treatment); Communicational; Relational<br>Annunziata: Informative; Assistance/care related, Relational; Psycho-emotional support; Material
CNQ-sf	Principal component analysis	Psychological; Health information; Physical and daily living; Patient care and support; Interpersonal communication needs
CSS-25	Exploratory factor analysis; Confirmatory factor analysis	Emotional wellbeing; Symptom burden and impact; Body image and healthy lifestyle; Health care team communication; Relationships and intimacy
BCNAS-32	Exploratory factor analysis	Psychological; Health system; Physical and daily living; Patient care and support; Sexuality; Logistics; Communication (spouse/partner); Communication (medical providers)
PCNQ V2	Principal component analysis	Part 1 (past issues and experiences): Specialist support; Anxiety and urgency; Confidence in treatment preparation and recovery; Knowledge and understanding; GP support; Informed choice; Other support for diagnosis and treatment<br>Part 2 (current issues and experiences): Role limitations; GP ongoing support; Impotence and sexual issues; Incontinence; Personal integration and control; Specialist ongoing support
YYFcore03	Exploratory factor analysis	Psychological distress; Logistics of medical care; Somatization (physical, practical, psychological, social problems)

Online Resource 11: Overall methodological quality appraisal

See ‘Online Resource clinimetrics’ spreadsheet - worksheet ‘Online Resource 11’.

[a]Original development was not accessible for CNQ-SF and not available for eHNA.

[b]In COSMIN, an item can be rated as *inadequate* if it has not been done, not been reported, or done inadequately.

Online Resource 12: GRADE levels of evidence with reasons

See ‘Online Resource clinimetrics’ spreadsheet – worksheet ‘Online Resource 12’.

Online Resource 13: Quality appraisal with reasons

See ‘Online Resource clinimetrics’ spreadsheet – worksheet ‘Online Resource 13’.

[a]As long as at least one of the ICCs/Kappa’s was >0.70, the instrument was rated as sufficient.

Online Resource 14: Instrument feasibility

**Instrument**	*Patient's comprehensibility*	*Clinician's comprehensibility*	*Type and ease of administration*	*Length of the instrument (items)*	*Completion time (mins)*	*Patient's required mental and physical ability level*
PNAP	Not reported	Not reported	Not reported	40	45	Not reported
SPARC	Items and response options were edited in response to patient feedback to improve understanding.	Not reported	Moderate - Interviewer-administered	46	15 to 20	Not reported
NA-ACP	86% found instrument clear and understandable. Flesch score of 76.9, understandable by 90% of people 25-64 years old.	Not reported	Easy - Self-administered	132	76	Understandable by 90% of people 25-64 years old.
NA-ALCP	Items and response options considered "understandable".	Not reported	Easy - Self-administered	38	Not reported	Not reported
PNPC	Not reported	Not reported	Easy - Self-administered	138	Not reported	Not reported
PNPC-sv	Not reported	Not reported	Easy - Self-administered	33	10	Not reported
SPEED	Not reported	Not reported	Moderate - Interviewer-administered	13	Not reported	Not reported
3LNQ	"Questions understood in same way by researcher and patient"	Not reported	Easy - Self-administered	35	Not reported	Not reported
Ndiok	Not reported	Not reported	Moderate - Interviewer-administered	104	Not reported	Not reported
PCNA-EAV	Considered by clinicians to be comprehensible for target population.	Not reported	Moderate - Interviewer-administered	116	40	Not reported
CaNDI	5.5 reading grade level calculated by Flesch-Kincaid.	Not reported	Easy - Self-administered	39	8	Understandable by >90% of people 25-64 years old.
CNAT	Not reported	Not reported	Moderate - Interviewer-administered	59	Not reported	Not reported
PNI	Not reported	Not reported	Easy - Self-administered	48	Not reported	Not reported

**Instrument**	*Patient's comprehensibility*	*Clinician's comprehensibility*	*Type and ease of administration*	*Length of the instrument (items)*	*Completion time (mins)*	*Patient's required mental and physical ability level*
eHNA	Not reported	Not reported	Easy - Self-administered	48	7	Not reported
SCNS-SF34	Favourable comprehensibility reported by patients. Can be understood by people with a seventh grade level of education.	Not reported	Easy - Self-administered	34	10	Not reported
SCNAT-IP	Item wording simplified to increase understanding.	Not reported	Moderate - Interviewer-administered	27 (26 and 1 open ended question)	15	Not reported
SCNS-ST9	Favourable comprehensibility reported by patients. Can be understood by people with a fourth to fifth grade reading level.	Not reported	Easy - Self-administered	9	Not reported	Not reported
SCNS-LF59	Favourable comprehensibility reported by patients. Can be understood by people with a fourth to fifth grade reading level.	Not reported	Easy - Self-administered	59	20	Not reported
NEQ	Good patient comprehensibility indicated by low missing scores and high satisfaction.	Not reported	Easy - Self-administered	23	5	Not reported
CNQ-sf	Not reported	Not reported	Easy - Self-administered	32	Not reported	Not reported
CSS-25	Not reported	Not reported	Easy - Self-administered	25	5 to 10	Not reported
BCNAS-32	Not reported	Not reported	Easy - Self-administered	32	Not reported	Not reported
PCNQ V2	Demonstrated to be understandable to the target population.	Not reported	Easy - Self-administered	69 (part 1: 39; part 2: 30)	30	Not reported
YYFcore03	Not reported	Not reported	Easy - Self-administered	31	Not reported	Not reported

**Instrument**	*Ease of standardisation*	*Ease of score calculation*	*Copyright*	*Cost of an instrument*	*Required equipment*	*Availability in different settings*	*Regulatory agency's requirement for approval*
PNAP	Not reported	Easy - scoring of individual items and total sum of item scores.	None reported (not reported)	None reported	Pen and printed instrument	Clinical and research	None reported
SPARC	Not reported	Easy - sum of item scores for each subscale and scoring of individual items.	None reported (downloadable from the web)	None	Pen and printed instrument	Clinical and research	None reported
NA-ACP	Not reported	Moderate - simple formula - need to transform scoring data so not applicable (=0), satisfied (=0), low need (=1), moderate need (=2), high need (=3), before summing items for each subscale.	None reported (email authors)	None reported	Pen and printed instrument	Clinical and research	None reported
NA-ALCP	Not reported	Moderate - simple formula - sum scores for items in each domain and divide by total number of items.	None reported (paper appendix)	None reported	Pen and printed instrument	Clinical and research	None reported
PNPC	Not reported	Easy - scoring of individual items/ sum of item scores for each subscale.	None reported (paper appendix)	None reported	Pen and printed instrument	Clinical and research	None reported
PNPC-sv	Not reported	Easy - scoring of individual items.	None reported (paper appendix)	None reported	Pen and printed instrument	Clinical and research	None reported
SPEED	Not reported	Easy - scoring of individual items.	None reported (within paper)	None reported	Pen and printed instrument	Clinical	None reported
3LNQ	Not reported	Moderate - simple formula, but subscales have different response options. Some question responses can be dichotomized into "unmet need" or "no need".	None reported (paper appendix)	None reported	Pen and printed instrument	Clinical and research	None reported
Ndiok	Not reported	Easy - scoring of individual items.	None reported (within paper)	None reported	Pen and printed instrument	Clinical and research	None reported

**Instrument**	*Ease of standardisation*	*Ease of score calculation*	*Copyright*	*Cost of an instrument*	*Required equipment*	*Availability in different settings*	*Regulatory agency's requirement for approval*
PCNA-EAV	Not reported	Easy - sum of item scores for each subscale and scoring of individual items.	Copyright 2011 by ProQuest LLC. (paper appendix)	None reported	Pen and printed instrument	Not reported	None reported
CaNDI	Not reported	Easy - total sum of item scores and sum of item scores for each subscale.	None reported (paper appendix)	None reported	Pen and printed instrument/ Touchscreen computer	Clinical and research	None reported
CNAT	Not reported	Easy - total sum of item scores.	None reported (within paper)	None reported	Pen and printed instrument	Clinical and research	None reported
PNI	Not reported	Easy - sum of item scores for each subscale.	None reported (within paper)	None reported	Pen and printed instrument	Clinical	None reported
eHNA	Not reported	Easy - scoring of individual items/ total sum of item scores.	Macmillan Cancer Support 2020 (sign up on Macmillan website)	None	Touchscreen computer	Clinical	None reported
SCNS-SF34	Complex - summing the individual items, subtracting m, and then multiplying the resulting value by 100/(m×(k-1)) for each subscale.	Complex - summing the individual items, subtracting m, and then multiplying the resulting value by 100/(m×(k-1)) for each subscale.	Supportive Care Needs Survey – Long Form 59 @ Centre for Health Research & Psycho-oncology, 2003, all right reserved (downloadable from the web)	None	Pen and printed instrument/ Touchscreen computer	Clinical and research	None reported
SCNAT-IP	Complex - (domain total score-m)*100/(m*[k-1]), in which m is the number of questions in a domain and k is the value of the maximum response for each item.	Easy - sum of item scores for each subscale/ total sum of item scores.	None reported (downloadable from SCNAT-IP website once completed training)	None	Pen and printed instrument/ Computer	Clinical	None reported

**Instrument**	*Ease of standardisation*	*Ease of score calculation*	*Copyright*	*Cost of an instrument*	*Required equipment*	*Availability in different settings*	*Regulatory agency's requirement for approval*
SCNS-ST9	Not reported	Easy - scoring of individual items.	Supportive Care Needs Survey – Long Form 59 @ Centre for Health Research & Psycho-oncology, 2003, all right reserved (within paper)	None	Pen and printed instrument/ Touchscreen computer	Clinical and research	None reported
SCNS-LF59	Complex - summing the individual items, subtracting m, and then multiplying the resulting value by 100/(m×(k-1)) for each subscale.	Complex - summing the individual items, subtracting m, and then multiplying the resulting value by 100/(m×(k-1)) for each subscale.	Supportive Care Needs Survey – Long Form 59 @ Centre for Health Research & Psycho-oncology, 2003, all right reserved (within paper)	None	Pen and printed instrument/ Touchscreen computer	Clinical and research	None reported
NEQ	Not reported	Easy - scoring of individual items/ sum of item scores for each subscale.	In the public domain (within paper)	None	Pen and printed instrument	Clinical and research	None reported
CNQ-sf	Not reported	Complex - scores converted to a score ranging from 0 to 100, but formula not reported. Sum of converted item scores for each subscale.	None reported (within paper)	None	Touchscreen computer	Clinical and research	None reported
CSS-25	Not reported	Moderate - total sum of item scores rated at or above two.	None reported (downloadable from the web)	None	Computer	Clinical, research and community	None reported
BCNAS-32	"Computed as recommended for the SCNS-LF59"	"Computed as recommended for the SCNS-LF59"	None reported (within paper)	None reported	Computer	Clinical	None reported
PCNQ V2	Not reported	Easy - scoring of individual items.	None reported (within paper)	None	Pen and printed instrument	Clinical	None reported
YYFcore03	Not reported	Easy - scoring of individual items.	None reported (within paper)	None reported	Touchpad	Clinical	None reported

Online Resource 15: Instrument interpretability

**Instrument**	*Distribution of scores in the study population*	*Percentage of missing items and percentage of missing total scores*	*Floor and ceiling effects*	*Scores and change scores available for relevant (sub)groups*	*Minimal important change (MIC) or minimal important difference (MID)*	*Information of response shift*
PNAP	Not reported.	Not reported.	Not reported.	Not reported.	Not reported.	Not reported.
SPARC	Not reported.	At least one question missing in 32 (51%) of 63 patients assessed.	Items exhibiting floor or ceiling effects were deleted or amended.	Not reported.	Not reported.	Not reported.
NA-ACP	Not reported.	Not reported.	Not reported.	Not reported.	Not reported.	Not reported.
NA-ALCP	Not reported.	<0.01%	Not reported.	Not reported.	Not reported.	Not reported.
PNPC	Yes - Frequency	Not reported.	Not applicable.	Not reported.	Not reported.	Not reported.
PNPC-sv	Yes - Frequency	Not reported.	Not applicable.	Not reported.	Not reported.	Not reported.
SPEED	Not reported	Not reported	Not reported.	Not reported.	Not reported.	Not reported.
3LNQ	Yes - Means	Not reported.	Not reported.	Not reported.	Not reported.	Not reported.
Ndiok	Yes - Frequency	Not reported.	Not applicable.	Not reported.	Not reported.	Not reported.
PCNA-EAV	Yes - Mean and SD	0-8% for most individual items. Two items missing 16%, one item missing 26%.	Not reported.	Yes - scores for subgroups (gender, age, location of residence, and ECOG)	Not reported.	Not reported.
CaNDI	Yes - Mean and SD; median and range	0-7% for individual items.	No ceiling effects were observed.	Yes - scores for subgroups (infusion and multidisciplinary clinic)	Not reported.	Not reported.
CNAT	Yes - Frequency & median	Not reported.	Considerable floor effect and little ceiling effect reported.	Yes - scores for subgroups (Karnofsky performance status, inpatient and outpatient, socio-demographics, treatment, stage, and cancer type)	Not reported.	Not reported.

**Instrument**	*Distribution of scores in the study population*	*Percentage of missing items and percentage of missing total scores*	*Floor and ceiling effects*	*Scores and change scores available for relevant (sub)groups*	*Minimal important change (MIC) or minimal important difference (MID)*	*Information of response shift*
PNI	Yes - Frequency	Not reported.	Not reported.	Yes - scores for subgroups (tumour type, critical moment, and global health status)	Not reported.	Not reported.
eHNA	Yes - Frequency	Not reported.	Not reported.	Yes - scores for subgroups (gender and curative)	Not reported.	Not reported.
SCNS-SF34	Yes - Mean and SD	Not reported.	No floor or ceiling effects observed.	Yes - scores for subgroups (remission or not)	Not reported.	Not reported.
SCNAT-IP	Yes - Mean, SD, Median and IQR	0.4-1.6% for each item.	Seven items with floor effects were removed. No ceiling effects observed.	Not reported.	Not reported.	Not reported.
SCNS-ST9	Yes - Frequency	Not reported.	Not reported.	Not reported.	Not reported.	Not reported.
SCNS-LF59	Not reported.	Not reported.	Not reported.	Not reported.	Not reported.	Not reported.
NEQ	Yes - Frequency	0-2% for each item.	Not applicable.	Yes - scores for subgroups (inpatients and outpatients)	Not reported.	Not reported.
CNQ-sf	Yes - Mean and SD	Not reported.	Not reported.	Yes - scores for subgroups (disease state, gender, ECOG performance status, treatment, fatigue, and age)	Not reported.	Not reported.
CSS-25	Yes - Mean and SD	<2% for each item.	Not reported.	Not reported.	Not reported.	Not reported.
BCNAS-32	Yes - Mean and SD	6-18% for all subscales.	Not reported.	Not reported.	Not reported.	Not reported.
PCNQ V2	Not reported.	43% of the sample missed at least one item.	Not reported.	Not reported.	Not reported.	Not reported.
YYFcore03	Not reported.	Not reported.	Not reported.	Not reported.	Not reported.	Not reported.
